# Novel Carbon-Based Magnetic Luminescent Nanocomposites for Multimodal Imaging

**DOI:** 10.3389/fchem.2020.00611

**Published:** 2020-07-24

**Authors:** Fangfang Liu, Xiaoming Mou, Jimei Song, Qin Li, Jinliang Liu

**Affiliations:** ^1^Shandong Peninsula Engineering Research Center of Comprehensive Brine Utilization, Weifang University of Science and Technology, Shouguang, China; ^2^School of Environmental and Chemical Engineering, Shanghai University, Shanghai, China

**Keywords:** superparamagnetism, upconversion nanoparticle, carbon, Fe_4_O_3_, nanocomposite

## Abstract

Multifunctional nanocomposites can combine multiple functions into a single nanosystem and thus have attracted extensive interest in various fields. The combination of magnetic and upconversion luminescent nanoparticles into one single nanoplatform, which have a good application in biomedical fields such as bio-magnetic separation, magnetic resonance imaging (MRI), and optical imaging, is highly desirable. Here we reported multifunctional nanocomposites by using hollow carbon sphere to integrate magnetic Fe_3_O_4_ and upconversion nanoparticles (UCNPs) into one nanosystem. The as-prepared UCNPs/Fe_3_O_4_@h-C have near-infrared (NIR) luminescence under 980 nm excitation and superparamagnetism. In addition, since the carbon layer can absorb NIR light and transfer it into heat with high efficiency, the nanocomposites can realize photo thermal (PT), upconversion luminescence (UCL) and MRI tri-mode imaging. The UCNPs/Fe_3_O_4_@h-C might be further utilized as a potential theranostic agent, including its in-depth monitoring through luminescent imaging and MRI diagnosis, as well as its direct use in tumors as a photothermal therapy (PTT) agent.

## Introduction

In recent years, multifunctional nanocomposite materials have attracted extensive interest because they can integrate multiple functionalities into one single nanosystem and thus endow them with great potential application in various areas (Gao et al., 2009; Liu Z. et al., 2011; Cheng et al., 2013; Jia et al., 2019; Sun et al., 2019; Liao et al., 2020). Among them, nanoparticles (NPs) with magnetism and luminescence have been extensively studied for their applications in biomedicine, for example, targeted enrichment and separation, magnetic targeted drug delivery, magnetic resonance imaging (MRI), biological luminescent probes (Cheng et al., 2016; Chen and Fu, 2018; Ding et al., 2018; Tang et al., 2018; Wang et al., 2018; Fu et al., 2019). Rare-earth doped upconversion luminescent nanomaterials (UCNPs) can emit high-energy photons of different wavelengths in visible or near-infrared (NIR) region by absorbing two or more low-energy photons in NIR region, this makes UCNPs have the prominent advantages over traditional phosphors in low autofluorescence background, shape emission bandwidths, good photochemical stability, and large tissue penetration depth (Xu et al., 2018; Zhu et al., 2018; Zhang et al., 2019). These unique properties proposed UCNPs as a new generation of optical materials in luminescent detections with high sensitivity, high resolution bioimaging, photodynamic therapies (PDT), and so on (Liu J. L. et al., 2011; Liu et al., 2017; Chen S. et al., 2018; Gu et al., 2019; Ma et al., 2019; Tang et al., 2019; Zhu et al., 2019). Magnetic nanoparticles are another kind of attractive materials for biomedicine because they can be fabricated using external magnetic fields. In consequence, the combination of magnetic nanoparticles and UCNPs into one nano-platform is highly pursued in biomedical applications, such as bio-magnetic separation, MRI and optical dual imaging, to improve the targeting efficiency and hyperthermia (Shen et al., 2010; Zhang et al., 2012; Zhong et al., 2012; Chen Z. X. et al., 2018).

Normally, UCNPs could be combined with magnetic materials in three ways. One is the SiO_2_-assisted synthetic strategy (Liu et al., 2008). Zhang et al. (2012) synthesized a kind of magnetic upconversion fluoride nanorattles Fe_3_O_4_@SiO_2_@α-NaYF_4_/Yb, Er (MUC-F-NR) through an ion-exchange method. The MUC-F-NR consisted a Fe_3_O_4_ magnetic core, a silica layer, a hollow space, and a NaYF_4_/Yb, Er shell. Further studies showed that the nanocomposites have low cytotoxicity, good cell imaging, and excellent tumor therapy efficacy *in vivo* when treated with an external magnetic field after loading with antitumor drug. The second is the cross-linker anchoring method. Shen et al. (2010) introduced 11-mercaptoundecanoic acid (MUA) or 1,10-decanedicarboxylic acid (DDA) as the crucial crosslinker to immobilize Fe_3_O_4_ NPs onto the surface of UCNPs, and synthesized the Fe_3_O_4_/NaYF_4_:Yb,Er hetero-NPs. These NPs could be well dispersed in water after ligand ozonolysis treatment, and may also acted as probes for biological imaging. The third is the seed-induced growth method (Zhong et al., 2012; Cheng et al., 2016; Qin et al., 2016). Zhong et al. (2012) first reported a seed-growth procedure to synthesize monodisperse core–shell nanoparticles Fe_3_O_4_@NaGdF_4_:Yb/Er@NaGdF_4_:Yb/Er. The Fe_3_O_4_ core enables the nanocrystals with superparamagnetic property, while the NaGdF_4_:Yb/Er outer shell markedly enhances the upconversion emission intensity. In spite that carbon nanomaterials have been extensively studied, the combination of carbon nanomaterials, UCNPs and Fe_3_O_4_ together to produce nanocomposites has been rarely reported. Zhu et al. (2016) constructed a carbon-coated nanocomposite, NaLuF_4_:Yb,Er@NaLuF_4_@Carbon (csUCNP@C), which can be used as a real-time monitor of the microscopic temperature in PTT. They found that the microscopic temperatures of the photothermal material upon irradiation were high enough to destroy cancer cells, while the lesions remained low enough to avoid the normal tissue from damage. Liu X. H. et al. (2011) coated a hydrophilic carbon layer on hydrophobic NaGdF_4_:Yb, Er nanocrystals to synthesize the core–shell structured NaGdF_4_:Yb,Er@Carbon nanocomposites. The prepared nanocomposites were with a uniform size of 25 nm and strong upconversion fluorescence, which can be potentially used as cell-imaging probes. They further developed NaYF_4_:Yb,Tm@C@CdS nanoparticles by depositing CdS on carbon-modified NaYF_4_:Yb,Tm nanocrystals. The nanocomposites showed good photocatalytic activity due to the efficient energy transfer from NIR light to ultraviolet-visible (UV) light of the NaYF_4_:Yb, Tm and the high adsorption ability of the carbon shell (Tou et al., 2016). We have combined Fe_3_O_4_ and UCNPs together by using a hollow carbon sphere to assemble a cancer theranostic agent. The nanoplatform can realize MRI-UCL dual-mode imaging and PTT for cancer with improved efficiency *in vivo* (Wang et al., 2019).

**Figure 1:**
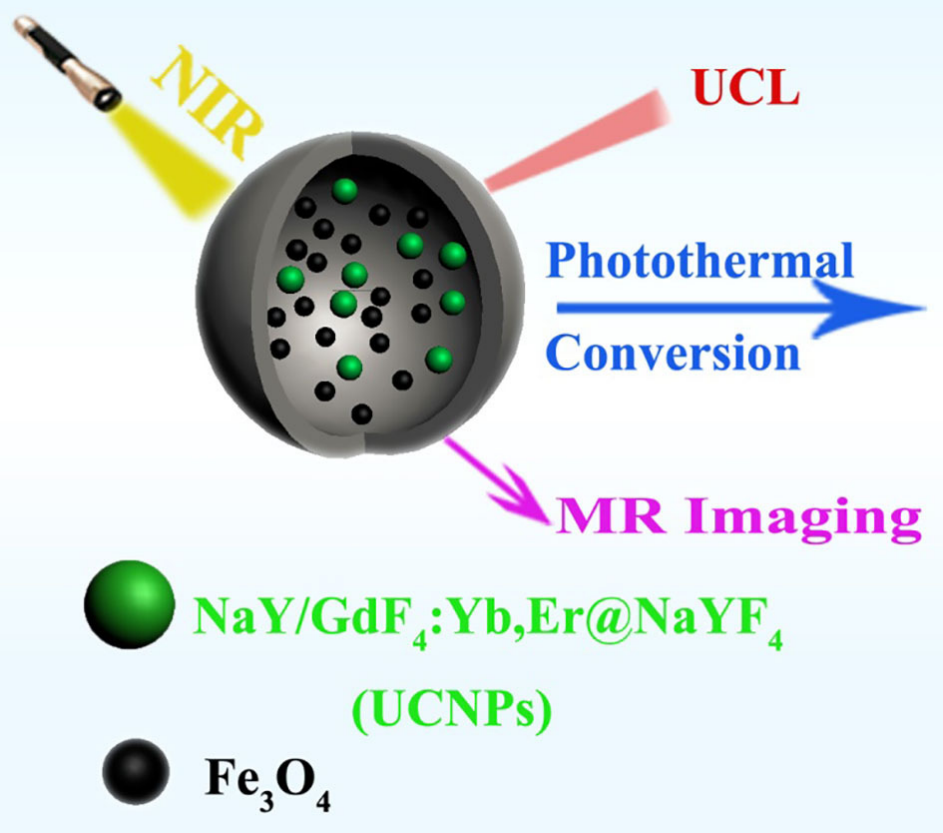
Schematic illustration of the synthesized UCNPs/Fe_3_O_4_@h-C nanocomposites with upconversion luminescence (UCL), magnetic resonance (MR), and photothermal (PT) imagings.

Inspired by the previous works mentioned above, we herein provided a kind of multifunctional nanocomposites which combine superparamagnetic Fe_3_O_4_, UCNPs and a hollow carbon sphere together. The synthesized nanocomposites were fabricated by introducing UCNPs and Fe_3_O_4_ into a cavity of hollow carbon sphere through a one-pot synthesis method, and the carbon shell wall acts as a protective shell, making it stable and free from the influence of the external environment. Since the carbon layer can convert the absorbed light into heat, the nanocomposites can realize photo thermal (PT), UCL, and MRI tri-mode imaging (Figure 1).

## Materials and Methods

### Materials

Cyclohexane (AR), anhydrous ethanol (AR), dichloromethane (CH_2_Cl_2_, AR), acetone (AR), iron (III) chloride hexaydrate (AR), hexamethylenetetramine (HMT, AR) were purchased from Sinopharm Chemical Reagent Co. China. Erbium (III) chloride hexaydrate (ErCl_3_·6H_2_O, 99.99%), gadolinium (III) chloride hexaydrate (GdCl_3_·6H_2_O, 99.99%), yttrium (III) chloride hexaydrate (YCl_3_·6H_2_O, 99.99%), ytterbium (III) chloride hexaydrate (YbCl_3_·6H_2_O, 99.99%), 1-octadecene (ODE), oleic acid (OA) were purchased from Sigma-Aldrich Co. Ltd. Sodium oleate (CP), n-hexane (AR), methanol (GC), sodium hydroxide (NaOH, GR), sodium oleate (NaOA, AR), ammonium fluoride (NH_4_F, GR), 2,4-dihydroxybenzoic acid (DA) were purchased from Shanghai Aladdin Chemistry Co., Ltd (Shanghai, China). All of the chemicals were used without further purification unless specified. A Milli-Q water system (18.2 MΩ•cm, Thermo Fisher) was used to provide the ultrapure water in the experiments.

### Synthesis of the Superparamagnetic Nanoparticles Fe_3_O_4_

The superparamagnetic nanoparticles Fe_3_O_4_ were synthesized according to a previous reported procedures (Park et al., 2004). Firstly, 5.4 g of FeCl_3_·6H_2_O and 18.3 g of NaOA were dissolved in a mixed solvent consisting of 40 mL of ethanol, 70 mL of hexane, and 30 mL of deionized water. The mixture was heated to 70°C and kept for 4 hours. After that, the upper organic layer containing iron oleate complex was washed three times with 15 mL of deionized water. After the hexane was evaporated off, the product iron oleate complex was obtained in a waxy solid form. Secondly, 4.5 g of the above synthesized iron oleate complexes and 0.7125 g of OA were dissolved in 32 mL of ODE. The reaction mixture was heated to 320°C and kept at this temperature for 30 min. When the reaction was completed, the solution was cooled to room temperature and 62.5 mL of ethanol was added. The nanocrystals Fe_3_O_4_ were precipitated and separated by centrifugation and dissolved in n-hexane for further usage.

### Synthesis of NaY/GdF_4_:Yb,Er

The solutions of YbCl_3_ (1 M, 400 μL), ErCl_3_ (0.1 M, 400 μL), GdCl_3_ (1 M, 500 μL), and YCl_3_ (1 M, 1,100 μL) were mixed together and heated to 110°C to evaporate the water. After cooling to room temperature, OA (12.0 mL) and ODE (30.0 mL) were added and the mixture was heated to 150°C to make the solid dissolved completely. Next, 20.0 mL of NaOH (0.2 g) and NH_4_F (0.3 g) methanol solution was added and then the reaction solution was heated to 60°C and kept for 30 min. After the evaporating of the methanol under vacuum, the solution was heated to 300°C and maintained for 1 h in an argon (Ar) atmosphere. Then, the solution was added with equal volume of acetone, the products were precipitated and collected by centrifugation and washed three times with the acetone/cyclohexane solution.

### Synthesis of NaY/GdF_4_:Yb,Er@NaYF_4_ (Denoted as UCNPs)

Eight hundred microliter of GdCl_3_ (1 M) was heated to 110°C until the water was evaporated completely, then OA (12.0 mL) and ODE (30.0 mL) were added and the mixture was heated to 150°C to make the solid dissolved completely. Next, 5 mL of cyclohexane solution containing the obtained NaY/GdF_4_:Yb was added and heated to evaporate the cyclohexane completely. After that, 20.0 mL of NaOH (0.2 g) and NH_4_F (0.3 g) methanol solution was added and the temperature was raised to 60°C to evaporate the methanol under vacuum. The next steps were similar to the process of the synthesis of NaY/GdF_4_:Yb mentioned above to gain the final product NaY/GdF_4_:Yb,Er@NaYF_4_, denoted as UCNPs.

### Synthesis of Carbon-Based Magnetic Luminescent Nanocomposites (UCNPs/Fe_3_O_4_@h-C)

Four hundred microliter of hexane solution containing 50 mg of UCNPs and 70 mg of Fe_3_O_4_ was added into 5 mL of water solution containing 100 mg of NaOA, the mixture was kept under ultrasonic for 10 min to form a water-in-oil emulsion. After the removal of hexane by evaporation at 50°C, the solution was transfer to a 150 mL reactor containing 0.3853 g of DA and 0.0876 g of HMT with 95 mL of deionized water. The mixture was rapidly heated to 160°C and kept for 4 h. The solid was obtained by centrifugation (8,000 rpm, 10 min), washed for 3 times with deionized water, dried at 60°C for 6 h and then heated to 500°C for 2 h under H_2_/Ar atmosphere (5%/95%, V/V). The final product is obtained and denoted as UCNPs/Fe_3_O_4_@h-C.

### Characterization

The morphologies of the samples were characterized by transmission electron microscopy (JEM-2010F, JEOL). Fourier transform infrared (FTIR) spectra were measured with Nicolet AVATAR370 FTIR spectroscopy. Upconversion luminescent spectra and luminescence decay curves were acquired by Edinburgh FS5 fluorescent spectroscope with a 0–2 W adjustable 980 nm continuous wave laser. Zeta potentials were measured by Malvern Zetasizer Nano ZSE. The magnetic property of the nanoparticles was performed using a Vibrating Sample Magnetometer (7407, lakeshore) at 298 K. The concentrations of Fe^3+^ were determined by inductively coupled plasma atomic emission spectroscopy (ICP-AES). The MRI property was measured using a SIMENS 3T MR scanner (MAGNETOM Trio Tim). The *r*_2_ relaxivity was obtained by linear fitting of the 1/*T*_2_ relaxation time (s^−1^) vs. the Fe^3+^ concentration (mM).

### Photothermal Performance Test of UCNPs/Fe_3_O_4_@h-C Nanocomposites

0.5 mL of UCNPs/Fe_3_O_4_@h-C of different concentrations (50, 100, 200, and 400 μg mL^−1^, respectively) dispersed in deionized water, fetal bovine serum (FBS) with different pH value (4, 6, 8), Dulbecco's Modified Eagle's Medium (DMEM), were irradiate under an 808 nm NIR semiconductor laser. The actual output power was precisely calibrated to be 1.5 Wcm^−2^ by using an optical power meter. The temperatures were recorded by a thermocouple with an accuracy of 0.1°C every 20 s. All data were acquired from three independent experiments.

### The Photothermal Conversion Efficiency Test of UCNPs/Fe_3_O_4_@h-C Nanocomposites

According to some previous reports (Roper et al., 2007; Zhu et al., 2016; Wei et al., 2017), the photothermal conversion efficiency η of UCNPs/Fe_3_O_4_@h-C nanocomposites was calculated using the following Equations (1)–(3):

(1) $$\begin{array}{r} \eta \;=\;\frac{\text{hS}\left(T_{\max}-\;T_{surr}\right)\;-\;Q_{Dis}}{I\left(1\;-10^{-A_{808}}\right)} \end{array}$$

(2) $$\begin{array}{r} \tau_{s}\;=\;\frac{m_{D}C_{D}}{hS} \end{array}$$

(3) $$\begin{array}{r} t=-\tau_{s}ln\theta \end{array}$$

In Equation (1), *h* is heat transfer coefficient, *S* is the container surface area, *T*_*max*_ is the equilibrium temperature, *T*_*surr*_ is ambient temperature, *Q*_*Dis*_ is heat dissipated from light absorbed by the container itself, which is measured independently containing water without UCNPs/Fe_3_O_4_@h-C, *A*_808_ is the absorption intensity of UCNPs/Fe_3_O_4_@h-C at 808 nm. In Equation (2), τ_*s*_ is the sample system time constant, m_D_ and C_D_ are the mass and heat capacity of the solvent (water), respectively.

### Cell Cytotoxicity Assay *in vitro*

The cell viabilities were measured with a CCK-8 Kit. Human cervical carcinoma cells (HeLa cells) were used for *in vitro* cytotoxicity assay of UCNPs/Fe_3_O_4_@h-C nanocomposites. The cells were seeded in 96-well plates at a density of 8 × 10^3^ cells per well and incubated overnight in DMEM containing 10% (vol·vol^−1^) FBS, penicillin (100 μg mL^−1^), and streptomycin (100 μg mL^−1^) at 37°C with 5% CO_2_, then the culture medium was replaced by fresh DMEM containing different concentrations (50, 100, 150, 200 μg/mL) of UCNPs/Fe_3_O_4_@h-C for another 24 h. The cell viability of the control group was set as 100% and that of other experimental groups were calculated based on the formula: cell viability = (Abs_450nm_ of the treated group/Abs_450nm_ of the control group) ×100%.

## Results and Discussion

### Synthesis and Characterization of UCNPs/Fe_3_O_4_@h-C

**Figure 2:**
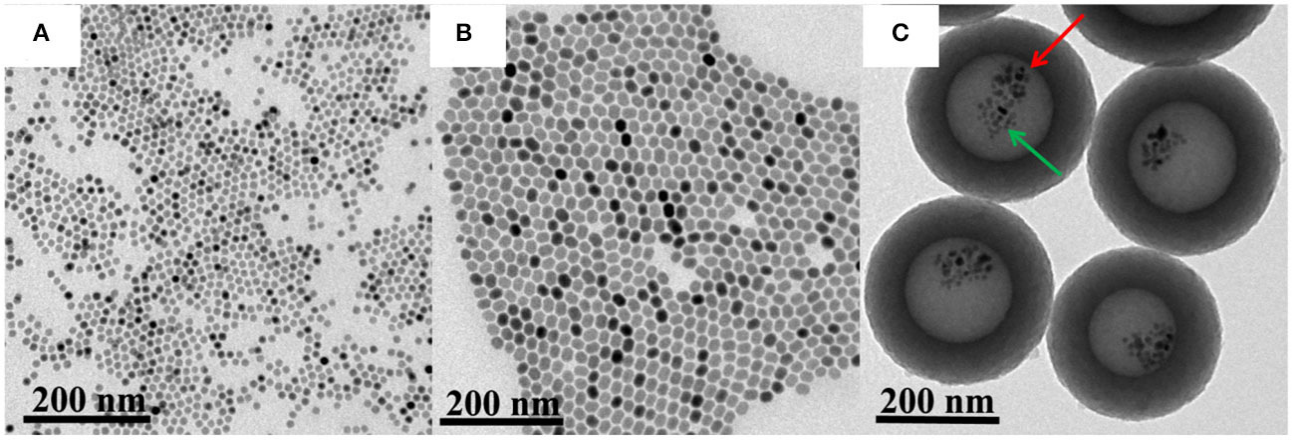
Transmission electron microscopy (TEM) images of Fe_3_O_4_ **(A)**, UCNPs **(B)**, and UCNPs/Fe_3_O_4_@h-C nanocomposites **(C)**. The red arrow indicates the UCNPs, and he green arrow indicates the Fe_3_O_4_.

Firstly, core-shell structured NaY/GdF_4_:Yb,Er@NaYF_4_ and superparamagnetic nanoparticles Fe_3_O_4_ were synthesized by a thermal decomposition method. Secondly, acted as the precursor of polymer layer, the interaction between DA and HMT made the solution acidic with a pH of 2.98, under which NaY/GdF_4_:Yb,Er@NaYF_4_ and Fe_3_O_4_ nanoparticles were introduced by using NaOA as the surfactant and soft template to transfer oleate-capped NaY/GdF_4_:Yb,Er@NaYF_4_ and Fe_3_O_4_ nanoparticles into an aqueous phase. Under a hydrothermal condition, HMT is decomposed into NH_3_ and HCHO to produce cavity structure. Meanwhile, during the heating process, NaOA emulsion droplets are heated and expanded, making the cavity volume grow larger. Finally, the temperature of the mixture was raised to 500°C and kept for 2 h under the atmosphere of Ar (95%) and H_2_ (5%) to generate the carbon shell from DA reduction (Sun et al., 2013). As shown in Figure 2A, the Fe_3_O_4_ has monodisperse morphology, and the particle size is about 12 nm calculated from the TEM images using ImageJ software (Figure S1a, Supplementary Information). In order to improve the luminescence intensity of NaY/GdF_4_:Yb,Er, a shell NaYF_4_ was coated on its outer layer to obtain the core-shell structure upconversion nanoparticles NaY/GdF_4_:Yb,Er@NaYF_4_ (denoted as UCNPs), as shown in Figure 2B. UCNPs had uniform morphology, good dispersion, and the particle sizes ranging from 18 to 21 nm (Figure S1b, Supplementary Information). The carbon-based multifunctional nanocomposites were finally obtained by means of the hollow carbon sphere to make Fe_3_O_4_ and UCNPs locate in the hollow cavity at the same time. As shown in Figure 2C, the synthesized UCNPs/Fe_3_O_4_@h-C nanocomposites have a hollow structure with a huge cavity about 160 nm in diameter, and the carbon layer was about 60–70 nm in thickness. The total size of the UCNPs/Fe_3_O_4_@h-C is calculated to be 280–300 nm Figure S1c, Supplementary Information). Moreover, we found that UCNPs and Fe_3_O_4_ existed simultaneously in the hollow cavity of the nanocomposites, UCNPs with a relatively large particle size (denoted by the red arrow) and Fe_3_O_4_ with a relatively small particle size (denoted by the green arrow) were observed at the same time. Compared with NaY/GdF_4_:Yb,Er@NaYF_4_ NPs, the particle size of UCNPs decreased when it entered into the hollow cavity of carbon sphere, which may be caused by OA-induced dissolution of the core-shell nanocrystals (Liu et al., 2016; Tang et al., 2019). The zeta potentials of NaY/GdF_4_:Yb,Er, UCNPs, and UCNPs/Fe_3_O_4_@h-C are −5.2 ± 1.13 mV, −4.0 ± 0.62 mV, and −7.5 ± 1.44 mV, respectively (Figure S2, Supplementary Information). Since the nanocomposites contain carbon materials, upconversion luminescence materials and magnetic materials, it is preliminarily believed that this nanosystem can realize photothermal, UCL and MRI trimode imaging simultaneously.

**Figure 3:**
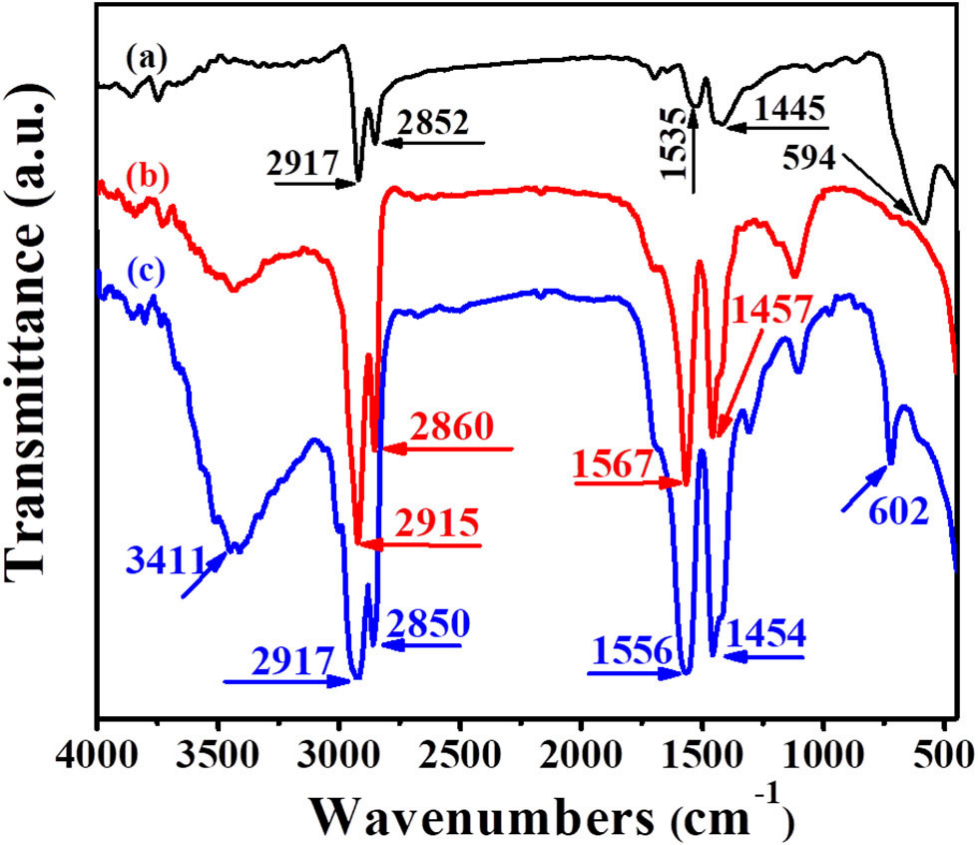
Fourier transform infrared (FTIR) spectra of Fe_3_O_4_ **(a)**, UCNPs **(b)**, and UCNPs/Fe_3_O_4_@h-C nanocomposites **(c)**.

The FTIR spectra of Fe_3_O_4_, UCNPs and UCNPs/Fe_3_O_4_@h-C are exhibited in Figures 3A–C, respectively. In Figure 3A, the peaks at 2,917 and 2,852 cm^−1^ correspond to the asymmetric and symmetric stretching vibrations of methylene (-CH_2_-) in the oleic acid chain, the peaks at 1,535 cm^−1^ correspond to the vibration of the unsaturated carbon-oxygen double bond (-C=O-) in the oleic acid, the peaks at 1,445 cm^−1^ correspond to the vibration of the submethyl (-CH), and the peaks at 594 cm^−1^ correspond to the Fe-O bond in Fe_3_O_4_. In Figure 3B, the asymmetric and symmetric stretching vibrations of methylene (-CH_2_-) of the UCNPs are at 2,915 and 2,860 cm^−1^, respectively, the vibration of the unsaturated carbon-oxygen double bond (-C=O-) and the submethyl (-CH) were corresponding to the peaks at 1,567 and 1,457 cm^−1^, respectively. In Figure 3C, similar peaks at 2,917, 2,850, 1,556, 1,454 cm^−1^ were observed, which assigned to the asymmetric and symmetric stretching vibrations of methylene (-CH_2_-) and the vibration of the unsaturated carbon-oxygen double bond (-C=O-). These peaks are attributed to the present of -CH_2_-CH_2_- and carbon-oxygen groups of hollow carbon nanospheres, as well as some contributions from the surface groups of UCNPs and Fe_3_O_4_ nanoparticles. Meanwhile, the peak at 602 cm^−1^ corresponding to the Fe-O bond suggested the existence of Fe_3_O_4_. The peak at 3,411 cm^−1^ corresponding to the stretching vibration of hydroxyl (OH) may due to the water molecules adsorbed and the hydroxyl on the surface of carbon spheres.

### Magnetic and UCL Properties of UCNPs/Fe_3_O_4_@h-C

**Figure 4:**
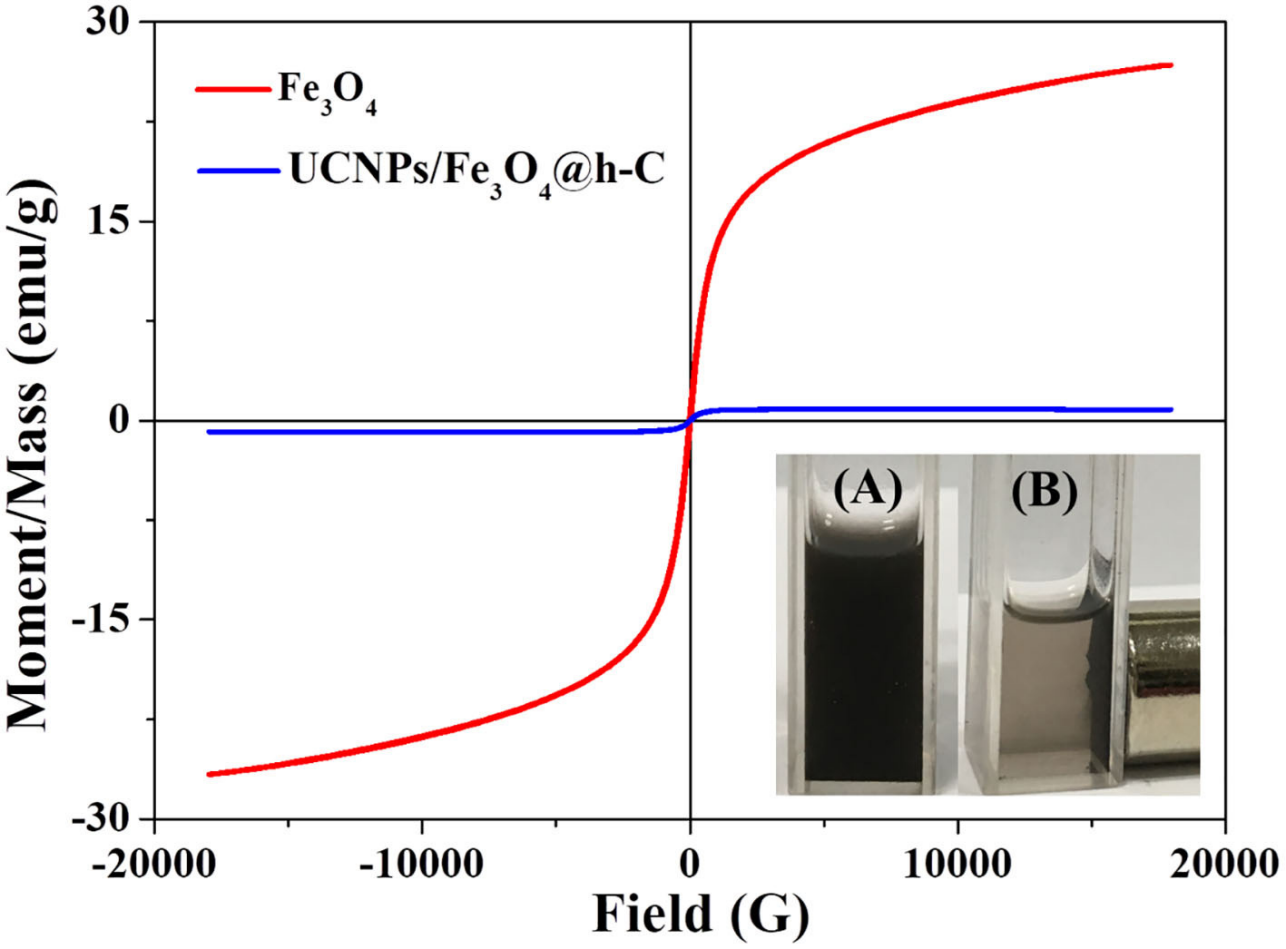
Magnetic hysteresis loops of Fe_3_O_4_ and UCNPs/Fe_3_O_4_@h-C nanocomposites at room temperature. Inset shows the digital photographs of UCNPs/Fe_3_O_4_@h-C nanocomposites without **(A)** or with **(B)** a commercial permanent magnet.

Due to the presence of Fe_3_O_4_ in the hollow nanocavity, the nanocomposites UCNPs/Fe_3_O_4_@h-C are expected to have good magnetic properties. The hysteresis loops of Fe_3_O_4_ (red) and UCNPs/Fe_3_O_4_@h-C (blue) are shown in Figure 4. Both Fe_3_O_4_ NPs and the UCNPs/Fe_3_O_4_@h-C nanocomposites showed superparamagnetic properties because no hysteresis was observed in the figures. The saturation magnetization of Fe_3_O_4_ is 26.68 emu/g. When Fe_3_O_4_ NPs and UCNPs were encapsulated into the hollow carbon cavity together, the saturation magnetization was markedly reduced to 0.87 emu/g, which was a little smaller than that of the samples (1.28 emu/g) prepared by Zhang et al. (2012). This may mainly because of the weight contribution from nonmagnetic NaY/GdF_4_:Yb,Er@NaYF_4_ materials and the hollow carbon materials. The magnetic separation ability of UCNPs/Fe_3_O_4_@h-C was further studied. Under the action of external magnetic field on one side of glass tube, these black nanoparticles are attracted by magnets in a short time, which proves the existence of magnetic Fe_3_O_4_ (Inset photos in Figure 4). Therefore, although the saturation magnetization value of UCNPs/Fe_3_O_4_@h-C is decreased, it still showed effective magnetic separation capability. These results demonstrated that the nanocomposites are expected for bioapplications in magnetic enrichment or separation.

**Figure 5:**
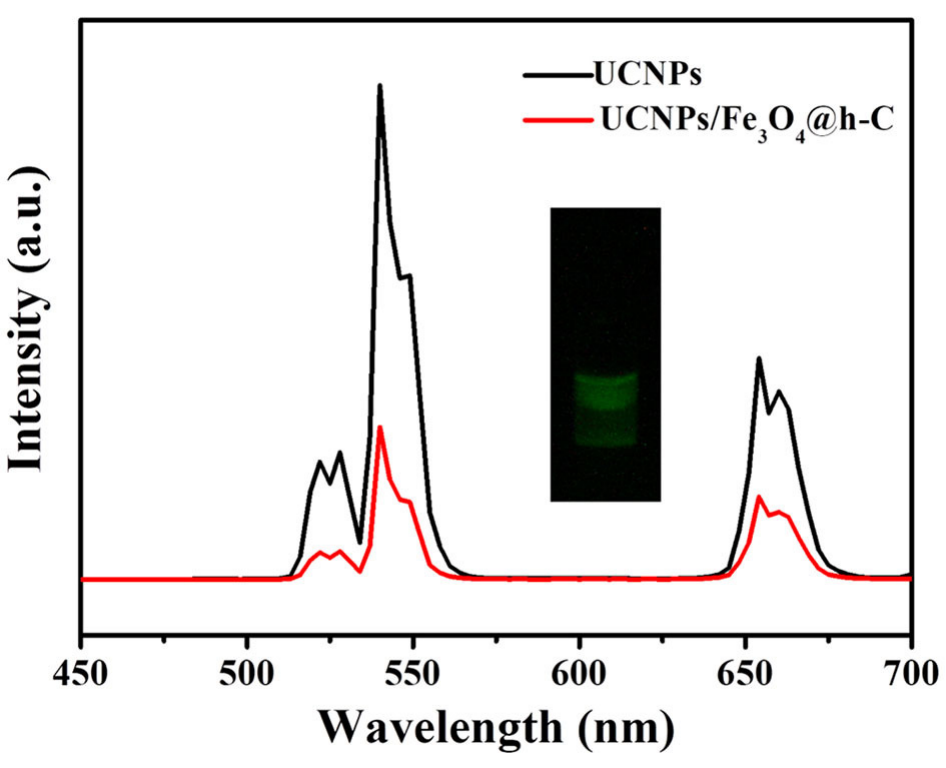
Upconversion luminescence spectra of UCNPs and UCNPs/Fe_3_O_4_@h-C nanocomposites. Inset shows the digital photograph of UCNPs/Fe_3_O_4_@h-C nanocomposites under 980 nm laser irradiation.

The upconversion luminescence spectra of NaY/GdF_4_:Yb,Er@NaYF_4_ and UCNPs/Fe_3_O_4_@h-C nanocomposites were measured and shown in Figure 5. When excited by a 980 nm laser, both NaY/GdF_4_:Yb,Er@NaYF_4_ and UCNPs/Fe_3_O_4_@h-C exhibited three independent characteristic peaks located at about 524, 545 and 654 nm, they were assigned to the ^2^H_11/2_→^4^I_15/2_, ^4^S_3/2_→^4^I_15/2_ and ^4^F_9/2_→^4^I_15/2_ transitions of Er^3+^ ions, respectively. The characteristic emission peaks of UCNPs/Fe_3_O_4_@h-C nanoparticles are similar to those of NaY/GdF_4_:Yb,Er@NaYF_4_, suggesting that the doping of Fe_3_O_4_ NPs does not basically impeded the luminescent performance of NaY/GdF_4_:Yb,Er@NaYF_4_ despite some partial quenching caused by the black Fe_4_O_3_ and the hollow carbon layer. The visualized presentation of UCL can also be tested by the photo taken from a digital camera, as shown in inset of Figure 5, visible green luminescence can be obviously seen when UCNPs/Fe_3_O_4_@h-C nanocomposites is irradiated with 980 nm excitation light. The luminescence decay curves of Er^3+^: ^4^S_3/2_→^4^I_15/2_ transition in NaY/GdF_4_:Yb,Er, NaY/GdF_4_:Yb,Er@NaYF_4_, and UCNPs/Fe_3_O_4_@h-C under 980 nm excitation was shown in Figure S3, and the lifetimes were calculated as 0.04, 0.46, and 0.02 ms for NaY/GdF_4_:Yb,Er, NaY/GdF_4_:Yb,Er@NaYF_4_, and UCNPs/Fe_3_O_4_@h-C, respectively. These results indicate that the obtained nanocomposites can retain the upconversion luminescence properties from UCNPs, which can be exploited in optical imaging *in vivo*.

### *In vitro* Photothermal Effect and MR Imaging of UCNPs/Fe_3_O_4_@h-C

**Figure 6:**
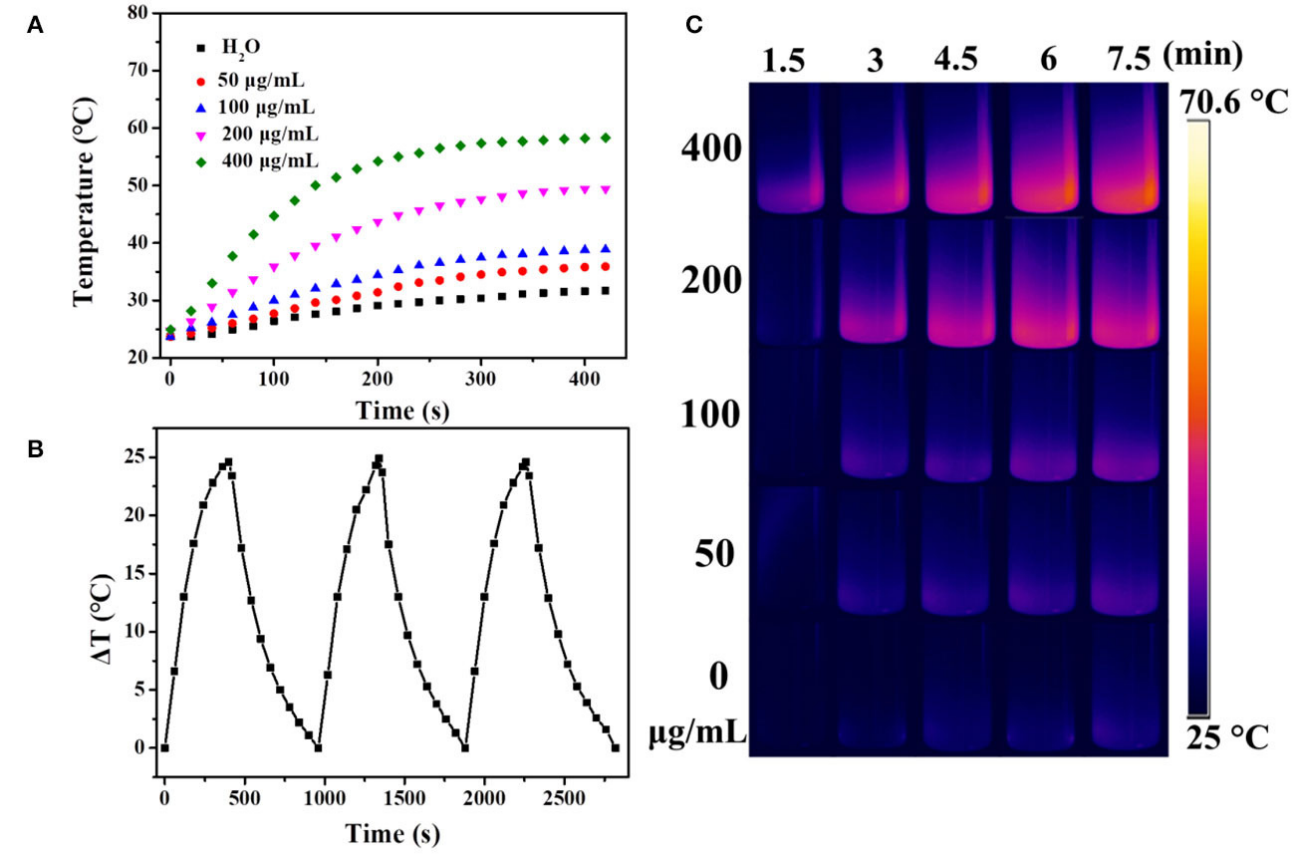
**(A)** The temperature changes of water, UCNPs/Fe_3_O_4_@h-C nanocomposites at different concentrations (50, 100, 200, 400 μg/mL, respectively) as a function of time under 808 nm laser irradiation at a power density of 1.5 W cm^−2^. **(B)** The temperature changes of UCNPs/Fe_3_O_4_@h-C nanocomposites during the three photothermal cycles. **(C)** Infrared thermal images of the different concentrations of UCNPs/Fe_3_O_4_@h-C nanocomposites at the time points of 1.5, 3, 4.5, 6, and 7.5 min under 808 nm laser irradiation (1.5 W cm^−2^).

**Figure 7:**
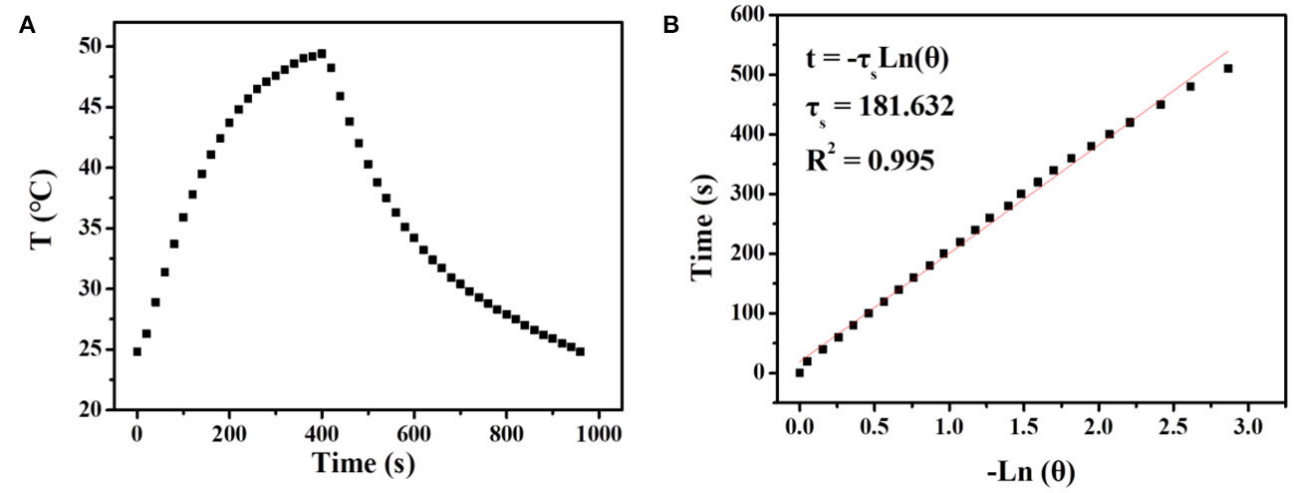
**(A)** The temperature changes of UCNPs/Fe_3_O_4_@h-C nanocomposites (200 μg mL^−1^) after 808 nm laser irradiation (1.5 Wcm^−2^) for 7 min, following the laser being turned off. **(B)** Time constant for heat transfer from the system calculated to be τ_s_ = 181.6 s by using the linear time data from the cooling period (after 420 s) vs. the negative natural logarithm of the driving force temperature, which is obtained from the cooling profile in Figure 6A.

It is well known that carbon materials have good heat-absorbing properties and can convert the absorbed NIR light into heat efficiently. Herein, the temperature changes of UCNPs/Fe_3_O_4_@h-C solution at different concentrations under continuous laser irradiation at 808 nm were measured to evaluate the photothermal conversion of the nanocomposites. As indicated in Figure 6, the temperature of the solution increased by 12.2, 15.2, 25.7, and 34.6°C after 7 min of illumination when the concentrations of the sample were 50, 100, 200, and 400 μg/mL, respectively. Compared with the control group of pure water, the temperature only increased by 8°C under the same conditions (Figure 6A). The photothermal conversions of the nanocomposites in different solutions were also evaluated. As shown in Figure S4 in Supplementary Information, similar trends of temperature changes were observed when UCNPs/Fe_3_O_4_@h-C dispersed in FBS with different pH value (4, 6, 8), or cell culture medium DMEM, which indicated that the solvents had little influence on the photothermal effect of UCNPs/Fe_3_O_4_@h-C composite. We further tested the thermal stability of UCNPs/Fe_3_O_4_@h-C nanocomposites. The sample (200 μg/mL) was irradiated with 808 nm laser for 7 min and then cooled naturally to room temperature. As shown in Figure 6B, the temperature changes of the three heating and cooling experiments are similar, indicating that the samples have good thermal stability. Furthermore, the infrared thermal images of UCNPs/Fe_3_O_4_@h-C with different concentrations at the time points of 1.5, 3, 4.5, 6, and 7.5 min were recorded. As shown in Figure 6C, the temperatures of the UCNPs/Fe_3_O_4_@h-C nanocomposites increased rapidly with the increase of concentration from 0 to 400 μg/mL. In addition, time constant of heat transfer from this system is determined to be τ_*s*_ = 181.6 s, and the photothermal conversion efficiency (η) was calculated to be 24.07% from the obtained data (Figures 7A,B). Thus, the nanocomposites with a good photothermal conversion effect and good photothermal stability may become a promising photothermal imaging and photothermal treatment agent for tumors.

**Figure 8:**
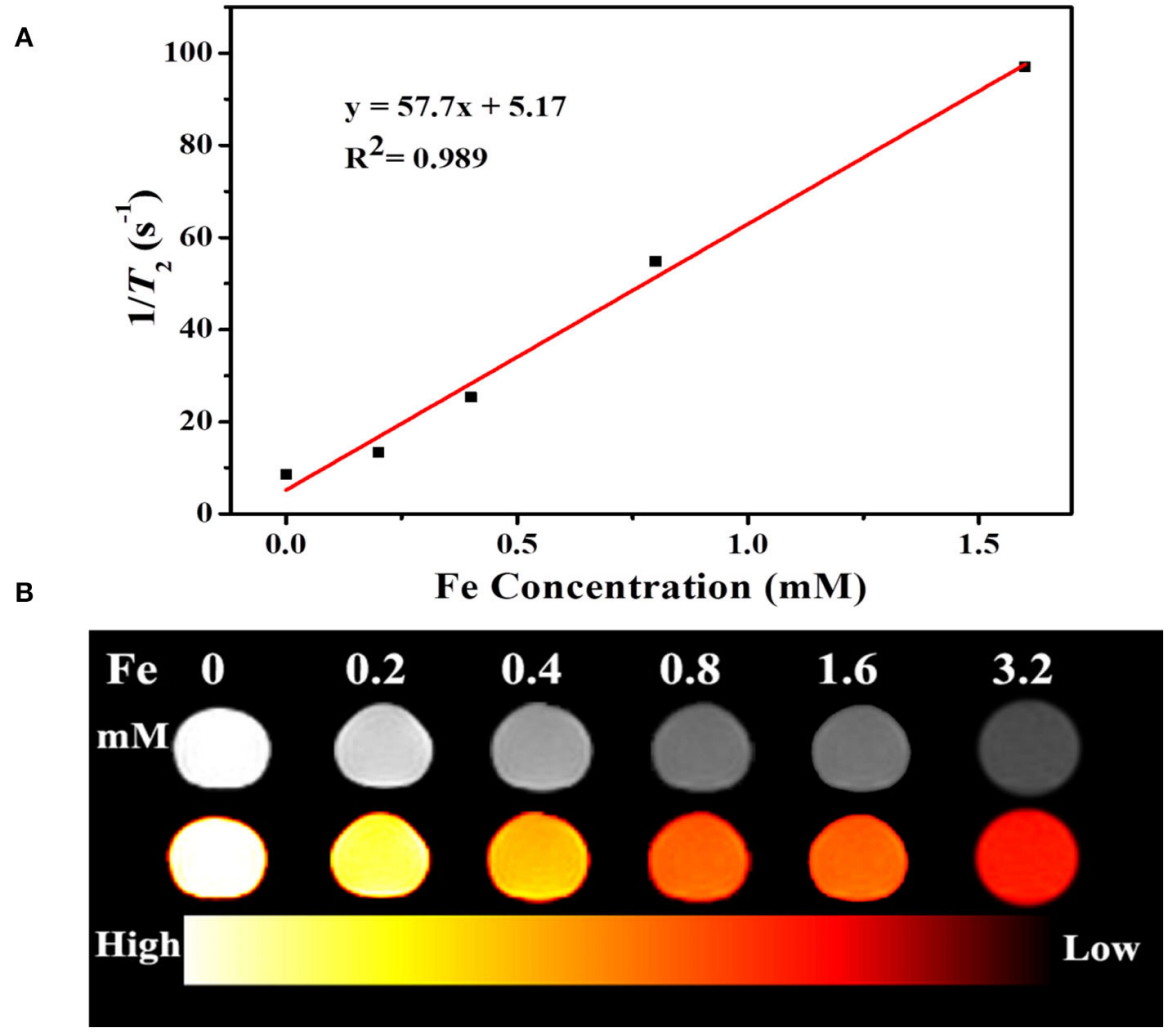
**(A)** Relaxation rate r_2_ (1/T_2_) plotted against the different Fe^3+^ concentrations of UCNPs/Fe_3_O_4_@h-C nanocomposites. **(B)** T_2_-weighted and color-mapped magnetic resonance (MR) images for various Fe^3+^ concentrations of UCNPs/Fe_3_O_4_@h-C nanocomposites.

Fe_3_O_4_ is well known as a contrast agent for MRI technique, which widely applied for noninvasive biological tissues imaging with high organ resolution. Figure 8A shows relaxation rate r_2_ (1/T_2_) plotted against the different Fe^3+^ concentrations of UCNPs/Fe_3_O_4_@h-C nanocomposites. The r_2_ value of UCNPs/Fe_3_O_4_@h-C is calculated by the linear curve with a slope of 57.7 mM^−1^S^−1^, indicated that the UCNPs/Fe_3_O_4_@h-C nanocomposites have potential applications in MRI as T_2_-weighted contrast agents. As shown in Figure 8B, the signal intensities decrease gradually with the increase of Fe^3+^ concentration, which can be seen vividly in a color mapped images.

### *In vitro* Cytotoxicity Assay of UCNPs/Fe_3_O_4_@h-C

The cytotoxicity of UCNPs/Fe_3_O_4_@h-C nanocomposites in HeLa cells was evaluated by using CCK-8 assay. As shown in Figure S5 in Supplementary Information, the cell viabilities were over 90% at all tested concentrations from 50 to 200 μg/mL, suggesting that UCNPs/Fe_3_O_4_@h-C has excellent biocompatibility.

## Conclusions

In conclusion, monodisperse, multifunctional UCNPs/Fe_3_O_4_@h-C nanocomposites have been successfully prepared. The NaY/GdF_4_:Yb,Er@NaYF_4_ UCNPs of the nanocomposites can convert the absorbed 980 nm NIR light into visible luminescence for UCL imaging, the carbon layer of the nanocomposites can convert the absorbed 808 nm NIR light into heat to realize photothermal imaging. Furthermore, the r_2_ value of UCNPs/Fe_3_O_4_@h-C (57.7 mM^−1^S^−1^) indicates the nanocomposites could be used as potential T_2_-weighted MRI contrast agents. To the best of our knowledge, this is the first report to load UCNPs and magnetic nanoparticle simultaneously in the cavity of the hollow carbon cavity to achieve upconversion luminescence, magnetic resonance, and photothermal imaging together.

## Data Availability Statement

The datasets generated for this study are available on request to the corresponding author.

## Author Contributions

The manuscript was written through contributions of all authors. All authors have given their approval of the final version of the manuscript.

## Conflict of Interest

The authors declare that the research was conducted in the absence of any commercial or financial relationships that could be construed as a potential conflict of interest.

## References

[B1] Chen S. Weitemier A. Z. Zeng X. He L. M. Wang X. Y. Tao Y. Q.. (2018). Near-infrared deep brain stimulation via upconversion nanoparticle-mediated optogenetics. Science 359, 679–683. 10.1126/science.aaq1144 29439241

[B2] Chen Z. X. Fu M. L. (2018). Recyclable magnetic Fe_3_O_4_@SiO_2_/beta-NaYE_4_:Yb^3+^,Tm-3(+)/TiO_2_ composites with NIR enhanced photocatalytic activity. Mater. Res. Bull. 107, 194–203. 10.1016/j.materresbull.2018.07.016

[B3] Chen Z. X. Fu M. L. Huang X. D. Yuan B. L. Yang J. C. E. (2018). Magnetic infrared responsive photocatalyst: fabrication, characterization, and photocatalytic performance of beta-NaYF_4_:Yb^3+^,Tm^3+^/TiO_2_/Fe_3_O_4_@SiO_2_ composite. Res. Chem. Intermediat. 44, 6369–6385. 10.1007/s11164-018-3495-9

[B4] Cheng L. Wang C. Ma X. X. Wang Q. L. Cheng Y. Wang H. (2013). Multifunctional upconversion nanoparticles for dual-modal imaging-guided stem cell therapy under remote magnetic control. Adv. Funct. Mater. 23, 272–280. 10.1002/adfm.201201733

[B5] Cheng Q. Guo H. X. Li Y. Liu S. X. Sui J. H. Cai W. (2016). A facile one-pot method to synthesize ultrasmall core-shell superparamagnetic and upconversion nanoparticles. J. Coll. Interface Sci. 475, 1–7. 10.1016/j.jcis.2016.04.040 27135942

[B6] Ding Y. D. Hong X. Zou P. Liu K. Cong T. Zhang H. (2018). Magnetic upconversion luminescent nanocomposites with small size and strong super-paramagnetism: polyelectrolyte-mediated multimagnetic-beads embedding. ACS Appl. Nano Mater. 1, 145–151. 10.1021/acsanm.7b00059

[B7] Fu S. W. Ding Y. D. Cong T. Yang X. G. Hong X. Yu B.. (2019). Multifunctional NaYF_4_:Yb,Er@PE3@Fe_3_O_4_ nanocomposites for magnetic-field-assisted upconversion imaging guided photothermal therapy of cancer cells. Dalton Trans. 48, 12850–12857. 10.1039/C9DT02329A 31393486

[B8] Gao J. H. Gu H. W. Xu B. (2009). Multifunctional magnetic nanoparticles: design, synthesis, and biomedical applications. Acc. Chem. Res. 42, 1097–1107. 10.1021/ar9000026 19476332

[B9] Gu Y. Y. Guo Z. Y. Yuan W. Kong M. Y. Liu Y. L. Liu Y. T. (2019). High-sensitivity imaging of time-domain near-infrared light transducer. Nat. Photonics 13:525 10.1038/s41566-019-0437-z

[B10] Jia T. Xu J. Dong S. He F. Zhong C. Yang G. (2019). Mesoporous cerium oxide-coated upconversion nanoparticles for tumor-responsive chemo-photodynamic therapy and bioimaging. Chem. Sci. 10, 8618–8633. 10.1039/C9SC01615E

[B11] Liao G. He F. Li Q. Zhong L. Zhao R. Che H. (2020). Emerging graphitic carbon nitride-based materials for biomedical applications. Progress Mater. Sci. 112:100666 10.1016/j.pmatsci.2020.100666

[B12] Liu D. Xu X. Du Y. Qin X. Zhang Y. Ma C.. (2016). Three-dimensional controlled growth of monodisperse sub-50 nm heterogeneous nanocrystals. Nat. Commun. 7:10254. 10.1038/ncomms10254 26743184 PMC4729871

[B13] Liu J. L. Liu Y. Liu Q. Li C. Y. Sun L. N. Li F. Y. (2011). Iridium(III) complex-coated nanosystem for ratiometric upconversion luminescence bioimaging of cyanide anions. J. Am. Chem. Soc. 133, 15276–15279. 10.1021/ja205907y 21892822

[B14] Liu X. H. Wang L. M. Wang Z. Y. Li Z. Q. (2011). Synthesis of biocompatible and luminescent NaGdF_4_:Yb,Er@Carbon nanoparticles in water-in-oil microemulsion. J. Mater. Res. 26, 82–87. 10.1557/jmr.2010.36

[B15] Liu Y. J. Lu Y. Q. Yang X. S. Zheng X. L. Wen S. H. Wang F.. (2017). Amplified stimulated emission in upconversion nanoparticles for super-resolution nanoscopy. Nature 543:229. 10.1038/nature21366 28225761

[B16] Liu Z. Sun L. N. Li F. Y. Liu Q. Shi L. Y. Zhang D. S. (2011). One-pot self-assembly of multifunctional mesoporous nanoprobes with magnetic nanoparticles and hydrophobic upconversion nanocrystals. J. Mater. Chem. 21, 17615–17618. 10.1039/c1jm13871e

[B17] Liu Z. Y. Yi G. S. Zhang H. T. Ding J. Zhang Y. W. Xue J. M. (2008). Monodisperse silica nanoparticles encapsulating upconversion fluorescent and superparamagnetic nanocrystals. Chem. Commun. 6, 694–696. 10.1039/B715402J 18478693

[B18] Ma Y. Q. Bao J. Zhang Y. W. Li Z. J. Zhou X. Y. Wan C. L.. (2019). Mammalian near-infrared image vision through injectable and self-powered retinal nanoantennae. Cell 177:243. 10.1016/j.cell.2019.01.038 30827682

[B19] Park J. An K. J. Hwang Y. S. Park J. G. Noh H. J. Kim J. Y.. (2004). Ultra-large-scale syntheses of monodisperse nanocrystals. Nat. Mater. 3, 891–895. 10.1038/nmat1251 15568032

[B20] Qin Z. L. Du S. N. Luo Y. Liao Z. J. Zuo F. Luo J. B. (2016). Hydrothermal synthesis of superparamagnetic and red luminescent bifunctional Fe_3_O_4_@Mn^2+^-doped NaYF_4_:Yb/Er core@shell monodisperse nanoparticles and their subsequent ligand exchange in water. Appl. Surf. Sci. 378, 174–180. 10.1016/j.apsusc.2016.03.219

[B21] Roper D. K. Ahn W. Hoepfner M. (2007). Microscale heat transfer transduced by surface plasmon resonant gold nanoparticles. J. Phys. Chem. C 111, 3636–3641. 10.1021/jp064341w 19011696 PMC2583113

[B22] Shen J. Sun L. D. Zhang Y. W. Yan C. H. (2010). Superparamagnetic and upconversion emitting Fe_3_O_4_/NaYF_4_:Yb,Er hetero-nanoparticles via a crosslinker anchoring strategy. Chem. Commun. 46, 5731–5733. 10.1039/c0cc00814a 20585692

[B23] Sun Q. Guo C. Z. Wang G. H. Li W. C. Bongard H. J. Lu A. H. (2013). Fabrication of magnetic yolk-shell nanocatalysts with spatially resolved functionalities and high activity for nitrobenzene hydrogenation. Chem. Eur. J. 19, 6217–6220. 10.1002/chem.201300307 23536472

[B24] Sun Q. He F. Bi H. Wang Z. Sun C. Li C. (2019). An intelligent nanoplatform for simultaneously controlled chemo-, photothermal, and photodynamic therapies mediated by a single NIR light. Chem. Eng. J. 362, 679–691. 10.1016/j.cej.2019.01.095

[B25] Tang M. Zhu X. Zhang Y. Zhang Z. Zhang Z. Mei Q.. (2019). Near-infrared excited orthogonal emissive upconversion nanoparticles for imaging-guided on-demand therapy. ACS Nano 13, 10405–10418. 10.1021/acsnano.9b04200 31448898

[B26] Tang Y. W. Liu H. Gao J. W. Liu X. Y. Gao X. Lu X. N.. (2018). Upconversion particle@Fe_3_O_4_@molecularly imprinted polymer with controllable shell thickness as high-performance fluorescent probe for sensing quinolones. Talanta 181, 95–103. 10.1016/j.talanta.2018.01.006 29426547

[B27] Tou M. J. Mei Y. Y. Bai S. Luo Z. G. Zhang Y. Li Z. Q. (2016). Depositing CdS nanoclusters on carbon-modified NaYF_4_:Yb,Tm upconversion nanocrystals for NIR-light enhanced photocatalysis. Nanoscale 8, 553–562. 10.1039/C5NR06806A 26647306

[B28] Wang J. X. Yao C. J. Shen B. Zhu X. H. Li Y. Shi L. Y.. (2019). Upconversion-magnetic carbon sphere for near infrared light-triggered bioimaging and photothermal therapy. Theranostics 9, 608–619. 10.7150/thno.27952 30809296 PMC6376195

[B29] Wang X. Xu J. Yang D. Sun C. Sun Q. He F. (2018). Fe_3_O_4_@MIL-100(Fe)-UCNPs heterojunction photosensitizer: rational design and application in near infrared light mediated hypoxic tumor therapy. Chem. Eng. J. 354, 1141–1152. 10.1016/j.cej.2018.08.070

[B30] Wei R. Y. Xi W. S. Wang H. F. Liu J. L. Mayr T. Shi L. Y.. (2017). *In situ* crystal growth of gold nanocrystals on upconversion nanoparticles for synergistic chemo-photothermal therapy. Nanoscale 9, 12885–12896. 10.1039/C7NR02280H 28650053

[B31] Xu M. Zou X. M. Su Q. Q. Yuan W. Cao C. Wang Q. H.. (2018). Ratiometric nanothermometer *in vivo* based on triplet sensitized upconversion. Nat. Commun. 9:7. 10.1038/s41467-018-05160-1 30002372 PMC6043590

[B32] Zhang F. Braun G. B. Pallaoro A. Zhang Y. C. Shi Y. F. Cui D. X.. (2012). Mesoporous multifunctional upconversion luminescent and magnetic “nanorattle” materials for targeted chemotherapy. Nano Lett. 12, 61–67. 10.1021/nl202949y 22133237

[B33] Zhang Z. Shikha S. Liu J. Zhang J. Mei Q. Zhang Y. (2019). Upconversion nanoprobes: recent advances in sensing applications. Anal. Chem. 91, 548–568. 10.1021/acs.analchem.8b04049 30260218

[B34] Zhong C. N. Yang P. A. P. Li X. B. Li C. X. Wang D. Gai S. L. (2012). Monodisperse bifunctional Fe_3_O_4_@NaGdF_4_:Yb/Er@NaGdF_4_:Yb/Er core-shell nanoparticles. RSC Adv. 2, 3194–3197. 10.1039/c2ra20070h

[B35] Zhu X. Zhang J. Liu J. Zhang Y. (2019). Recent progress of rare-earth doped upconversion nanoparticles: synthesis, optimization, and applications. Adv. Sci. 6, 1–30. 10.1002/advs.201901358 31763145 PMC6865011

[B36] Zhu X. J. Feng W. Chang J. Tan Y. W. Li J. C. Chen M.. (2016). Temperature-feedback upconversion nanocomposite for accurate photothermal therapy at facile temperature. Nat. Commun. 7:10. 10.1038/ncomms10437 26842674 PMC4742858

[B37] Zhu X. J. Li J. C. Qiu X. C. Liu Y. Feng W. Li F. Y. (2018). Upconversion nanocomposite for programming combination cancer therapy by precise control of microscopic temperature. Nat. Commun. 9:11. 10.1038/s41467-018-04571-4 29872036 PMC5988832

