# Supplementary material for: Novel Carbon-Based Magnetic Luminescent Nanocomposites for Multimodal Imaging
Source: Front Chem. 2020 Jul 24;8:611. doi: 10.3389/fchem.2020.00611 (PMC7393243; doi:10.3389/fchem.2020.00611)
Supplement: Supplementary file 1 [file Data_Sheet_1.PDF]

Supplementary Information

Novel Carbon-based Magnetic Luminescent Nanocomposites for Multimodal Imaging

Fangfang Liu[1]*, Xiaoming Mou[2], Jimei Song[1], Qin Li[2], and Jinliang Liu[2]*

[1] Shandong Peninsula Engineering Research Center of Comprehensive Brine Utilization, Weifang University of Science and Technology, Shouguang, Shandong, 262700, China

[2] School of Environmental and Chemical Engineering, Shanghai University, 99 Shangda Road, 200444, Shanghai, China

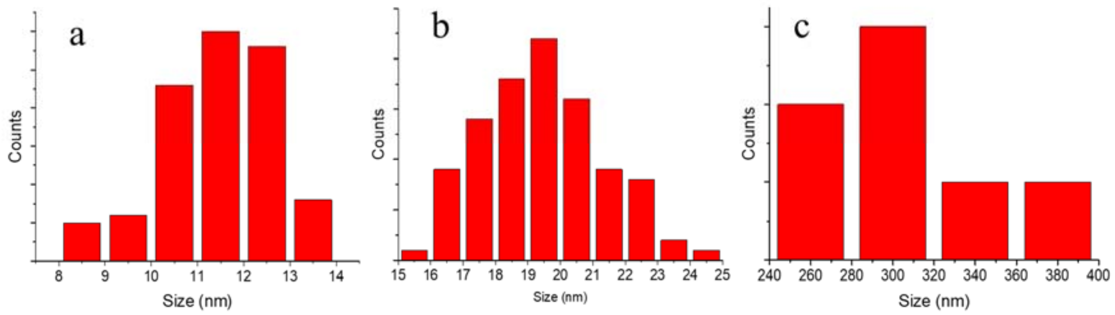

Figure S1. The statistical analysis for size values of Fe_3O_4 (a), UCNPs (b) and UCNPs/Fe_3O_4@h-C nanocomposites (c) using ImageJ software.

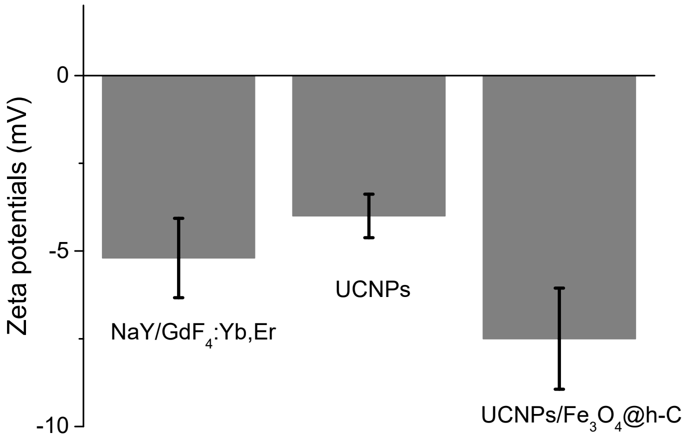

Figure S2. Zeta potentials of NaY/GdF_4:Yb,Er, UCNPs and UCNPs/Fe_3O_4@h-C.

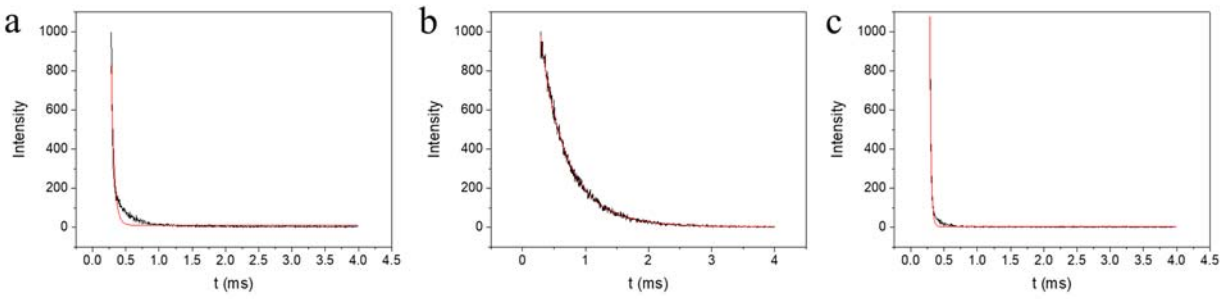

Figure S3. Luminescence decay curves of Er^{3+}: $^4S_{3/2} \rightarrow {}^4I_{15/2}$ transition in NaY/GdF_4:Yb,Er (a) NaY/GdF_4:Yb,Er@$NaYF_4$ (b) and UCNPs/Fe_3O_4@h-C (c) under 980 nm excitation.

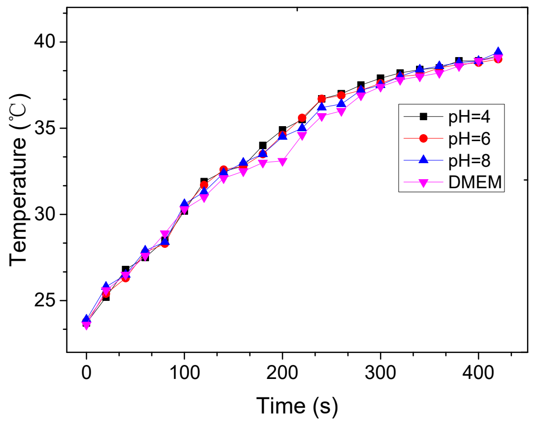

Figure S4. The temperature changes of UCNPs/Fe_3O_4@h-C nanocomposites (100 μg/mL) in different solutions (FBS with different pH value, or cell culture medium DMEM) as a function of time under 808 nm laser irradiation at a power density of 1.5 W cm^{-2}.

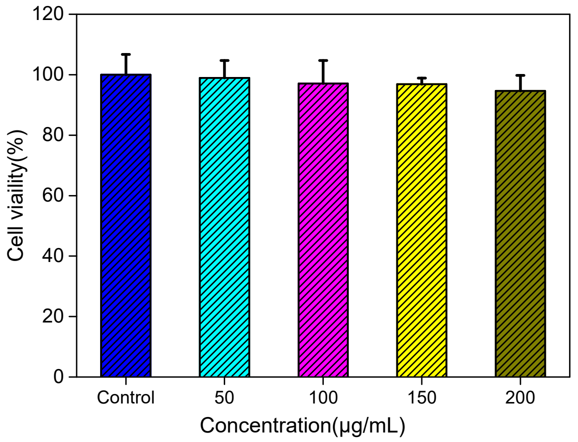

Figure S5. The cell viabilities of HeLa cells incubated with different concentrations of UCNPs/Fe_3O_4@h-C nanocomposites (50, 100, 150, 200 μg/mL, respectively) for 24 h.
